# Effects of Expected Progeny Difference and Feeding Systems on Carcass Characteristics in Hanwoo Steers

**DOI:** 10.3390/ani16121931

**Published:** 2026-06-22

**Authors:** Wonkyung Kim, Hyunjin Cho, Seongwon Seo

**Affiliations:** 1 Department of Animal Biosystem Sciences, Chungnam National University, Daejeon 34134, Republic of Korea; archikwk@daum.net (W.K.); chohyunjin0927@gmail.com (H.C.); 2 Cargill Animal Nutrition Korea, Seongnam 13630, Republic of Korea

**Keywords:** expected progeny difference, carcass traits, feeding system, total mixed fermented feed, Hanwoo steers, field data

## Abstract

This study evaluated the effects of expected progeny difference (EPD) grade and feeding system on carcass performance in Hanwoo steers using a large-scale field dataset collected under commercial production conditions. Records from 4561 steers (1466 fed total mixed fermented feed [TMF] and 3095 on a conventional separate-feeding system) across 269 farms in Korea from January 2023 to May 2025 were analyzed. Expected progeny difference grades for carcass weight (CWT), backfat thickness (BFT), ribeye area (REA), and marbling score (MBS) were classified A-D. Carcass performance significantly differed among EPD grades. Compared with grade D, grade A steers exhibited greater CWT (45.2 kg), less BFT (3.44 mm), greater REA (10.77 cm^2^), and greater MBS (1.57 units). Genetically superior animals reached slaughter age earlier. Steers fed TMF demonstrated higher CWT, BFT, REA, and MBS than conventionally fed steers. No significant interaction between EPD grade and feeding system was found for any carcass trait. These results indicate that EPD grades consistently predict carcass performance across different feeding environments, while TMF improves the absolute level of carcass traits. This large field dataset demonstrates that integrating Hanwoo EPD information with appropriate feeding management may support more efficient and profitable carcass production under commercial farm conditions.

## Simple Summary

Hanwoo is a Korean native cattle breed highly valued for its premium beef quality, and improving carcass traits such as meat yield and marbling, which are directly linked to farm profitability, is a central goal of the Hanwoo industry. This study examined the effects of genetic potential, measured by expected progeny difference (EPD) grades, and feeding systems on carcass performance in Hanwoo steers using large-scale commercial farm data. By analyzing production records from 4561 steers, we compared the effects of four distinct EPD grades alongside two feeding systems: a total mixed fermented feed (TMF) and a conventional separate feeding. The results showed that steers with the highest EPD grade produced significantly heavier carcasses with larger ribeye areas, increased marbling scores, and reduced backfat, and also reached their target slaughter age earlier. Additionally, steers fed TMF exhibited overall superior carcass performance compared to the conventional separate feeding system. No interaction between EPD grades and feeding systems was observed for any carcass trait. These findings suggest that integrating EPD information with appropriate feeding management can serve as a practical approach to supporting efficient and profitable beef production under commercial farm conditions.

## 1. Introduction

In the beef cattle production industry, continuous improvements in productivity have been driven by systematic advancements in genetic improvement and feeding management strategies. With recent progress in genetic evaluation technologies and the adoption of precision feeding practices, scientific approaches to improving cattle productivity have evolved beyond simple modifications of diet composition toward quantitatively elucidating the interactions between genetic potential and environmental factors [1,2].

Expected progeny difference (EPD) is a genetic evaluation index that statistically predicts the extent to which the progeny of a given sire are expected to differ from the progeny of an average parent for major carcass characteristics [3], including carcass weight (CWT), ribeye area (REA), backfat thickness (BFT), and marbling score (MBS). In the Korean Hanwoo industry, where carcass characteristics have a substantial impact on farm profitability, EPD has been widely utilized as a representative genetic evaluation tool to enhance selection efficiency and maximize economic value through the identification of genetically superior animals [4,5,6].

Previous studies have investigated the relationships between genetic evaluation indices including EPD, estimated breeding value (EBV), or genomic EBV (gEBV) and carcass characteristics, as well as the effects of feeding systems or nutritional levels on the phenotypic expression of genetic potential. Choi et al. [5] reported relatively high heritability estimates for MBS (h^2^ = 0.62 ± 0.07), supporting the practical contribution of EPD-based selection to improvements in carcass characteristics. Silva Neto et al. [2], in a comprehensive review of 116 beef and dairy cattle studies published between 1967 and 2023, reported that 83.62% of studies exhibited genetic correlations less than 0.80, indicating the presence of genotype × environment (G×E) interactions, whereby genetically superior animals tend to express their genetic potential more fully under optimal nutritional conditions. Furthermore, Gajaweera et al. [7] conducted a nutrigenomic study that analyzed relationships between gene expression and nutrients. The authors reported strong correlations between marbling-related gEBV and actual carcass phenotypes. However, despite an increase in dietary energy, particularly an increase in total digestible nutrients (TDN) of approximately 2 percentage points, this study observed no significant differences in the degree of genetic potential expression. These findings suggest that environmental modifications, such as high-energy feeding strategies, do not always result in predictable expression of genetic potential and that the effects may vary depending on the combination of genetic characteristics and feeding conditions.

Among various environmental factors, feeding systems are known to play a critical role in rumen fermentation dynamics, nutrient utilization efficiency, and patterns of growth and fat deposition. In Korea, the feeding systems commonly used for Hanwoo steers are generally classified into conventional separate feeding systems and total mixed ration (TMR) systems. Separate feeding has traditionally been the most widely adopted feeding system in the Hanwoo industry and involves feeding concentrates and forage separately. However, separate feeding may increase ruminal pH fluctuations due to temporal separation of concentrate and forage intake [8], and selective feeding behavior toward more palatable feeds may reduce the uniformity of nutrient intake and increase variation in nutrient supply among animals [9]. To overcome these limitations associated with separate feeding systems and to stabilize the rumen environment through uniform nutrient intake, the TMR system was introduced [10]. TMR consists of physically mixing concentrates, forages, and various additives in accordance with nutrient requirements, thereby reducing selective feeding and improving nutrient intake balance, digestibility, and overall productivity [10]. As a result, its adoption in the Hanwoo industry has steadily increased [11]. More recently, fermented TMR produced through anaerobic fermentation with lactic acid-producing bacteria (LAB) has gained increasing attention and adoption in the Hanwoo industry. Fermented TMR, also referred to as total mixed fermented feed (TMF), has been suggested to offer superior palatability, storage stability, feed hygiene, and intake uniformity compared with conventional TMR [12]. In addition, organic acids produced during fermentation stabilize microbial communities and further enhance starch and NDF digestibility as well as rumen fermentation stability [13,14].

Previous studies evaluating TMR or TMF feeding in Hanwoo steers have yielded inconsistent results, suggesting that the effects of mixed-ration feeding depend on diet composition, fermentation status, feeding period, and the traits evaluated. Chung et al. [15] reported that steers fed concentrates and forage separately showed greater growth performance, larger REA, and more desirable meat color than those fed TMR. In contrast, Lee et al. [12] found that fermented TMR increased dry matter and TDN intake, CWT, REA, and MBS, leading to improved net income despite higher feed costs. Park et al. [16] reported no marked differences in overall growth performance or carcass grade among feeding treatments, although TMF feeding affected selected blood metabolites and some meat quality-related traits. In addition, Lee, et al. [17] showed that TMR feeding altered chewing and rumination behavior compared with separate feeding, indicating that feed form can influence ingestion and rumination patterns. Earlier work by Cho et al. [18] also suggested that the practical value of TMR feeding in Hanwoo production may vary depending on production objectives and diet formulation. Collectively, these findings indicate that the advantages of TMR or TMF feeding in Hanwoo steers are not uniform and remain difficult to generalize across studies.

Furthermore, it remains unclear whether the genetic potential represented by EPD is consistently expressed across different feeding systems. In particular, integrated studies simultaneously considering genetic potential and feeding environment using large-scale field data from Hanwoo steers are limited. While studies addressing genetic factors or feeding environment independently have accumulated, systematic evaluations of the effects of G×E interactions on carcass characteristics remain relatively scarce.

Therefore, the present study was conducted to objectively assess the practical value and on-farm applicability of Hanwoo EPDs. Using data from 4561 Hanwoo steers, this study aimed to (1) quantitatively analyze the relationships between EPD and slaughter carcass characteristics and (2) evaluate whether these relationships differ according to feeding systems during the fattening period (separate feeding vs. TMF). The findings of this study are expected to provide fundamental information for enhancing the utilization of genetic evaluation and improving feeding management strategies in the Hanwoo industry.

## 2. Materials and Methods

### 2.1. Dataset

This study was conducted using production and carcass records from a total of 5037 Hanwoo steers raised on 269 farms in the Gyeongsang region of Korea between January 2023 and May 2025. The farms employed either a conventional feeding system, in which forage and concentrate mix were provided separately, or a TMF feeding system. Within each farm, animals were managed under the same feeding system. Diets for both groups were formulated based on the recommended average daily gain ranges for Hanwoo steers at each growth stage according to the Korean Feeding Standards for Hanwoo [19], targeting an overall mean ADG of approximately 0.8–0.9 kg/day across the entire production period to achieve a target slaughter age of 30 months. Detailed ingredient compositions of the experimental diets are presented in Table 1 and Table 2.

In both feeding systems, steers were fed twice daily in the morning and afternoon, and drinking water was provided ad libitum. All animals were slaughtered at officially certified Nonghyup livestock slaughterhouses in the Republic of Korea. The evaluated carcass traits, including CWT, BFT, REA, and MBS, were officially measured by certified graders from the Korea Institute for Animal Products Quality Evaluation using standardized instruments and established protocols. Subsequently, the data were obtained from official grading records based on the Korean carcass grading standards [20].

Only animals that were clinically healthy at the time of shipment and had complete pedigree, registration, and EPD information were included in the analysis. Of the 5037 initially collected records, 476 animals were excluded due to missing carcass or EPD data, early slaughter caused by disease or accidents, or suspected recording errors. Consequently, a total of 4561 steers remained in the dataset. Among these, 1466 were fed TMF, whereas 3095 were managed under a conventional feeding system.

### 2.2. Expected Progeny Difference Grades

Individual EPD values and grades at the time of analysis were obtained from the sire genetic evaluation reports provided by Korea Animal Improvement Association [21]. For each carcass trait, EPD values, defined as the expected genetic deviation of an individual’s progeny from the population mean, were obtained from the national Hanwoo genetic evaluation system.

With the EPD system, animals are ranked within the reference population for each trait and categorized into a four-tier percentile-based grading system (A, B, C, and D), where grade A represents the most favorable genetic merit and grade D the least favorable genetic merit for the corresponding trait [21]. Specifically, animals in the top 1–20% of the population were classified as grade A, those in the 20–45% range as grade B, those in the 45–70% range as grade C, and those in the 70–100% range as grade D. These EPD grades are updated twice annually (February and August) as progeny test data accumulate, and the EPD values of existing sires may change as additional records are incorporated.

### 2.3. Feeding Systems

For the TMR feeding system, TMF was produced through sequential steps, including preparation of a liquid inoculum, production of a primary fermented feed, and final mixing and fermentation of the TMF. The liquid inoculum was prepared by mixing 3.6 kg of powdered microbial culture (ASO Co., Ltd. KNU Research Center, Gyeongsangbuk-do, Korea) containing three lactic acid bacteria strains (Lactobacillus plantarum [9.0 × 10^11^ CFU/g], Lactobacillus acidophilus [9.8 × 10^10^ CFU/g], and Lactobacillus casei [6.0 × 10^11^ CFU/g]) and one yeast strain (Saccharomyces cerevisiae [3.0 × 10^10^ CFU/g]) with 125 g of glucose in 10 L of water. The mixture was then incubated at 30 °C for 24 h.

The primary fermented feed was prepared by adding the liquid inoculum to a mixture at an inoculum-to-mixture ratio of 1:9 (as-fed [AF] basis). The mixture consisted of 62.2% base concentrate mix, 18.7% corn DDGS, 7.9% rice bran, 4.6% soybean meal, and 6.7% cane molasses on a DM basis. The base concentrate mix consisted of corn grain (28.9%), soybean meal (6.9%), corn DDGS (15.0%), palm kernel meal (10.1%), corn gluten feed (20.8%), wheat bran (9.8%), rice bran (5.0%), cane molasses (1.8%), and other (1.7%) on a DM basis. After adjusting the moisture content of the mixture to approximately 43%, the mixture was sealed in vinyl ton bags and subjected to anaerobic fermentation at ambient temperature for at least 15 d. This resulting primary fermented feed was incorporated at 20% and 40% of the total formulation for the growing-phase TMF and fattening-phase TMF, respectively.

**Figure 1:**
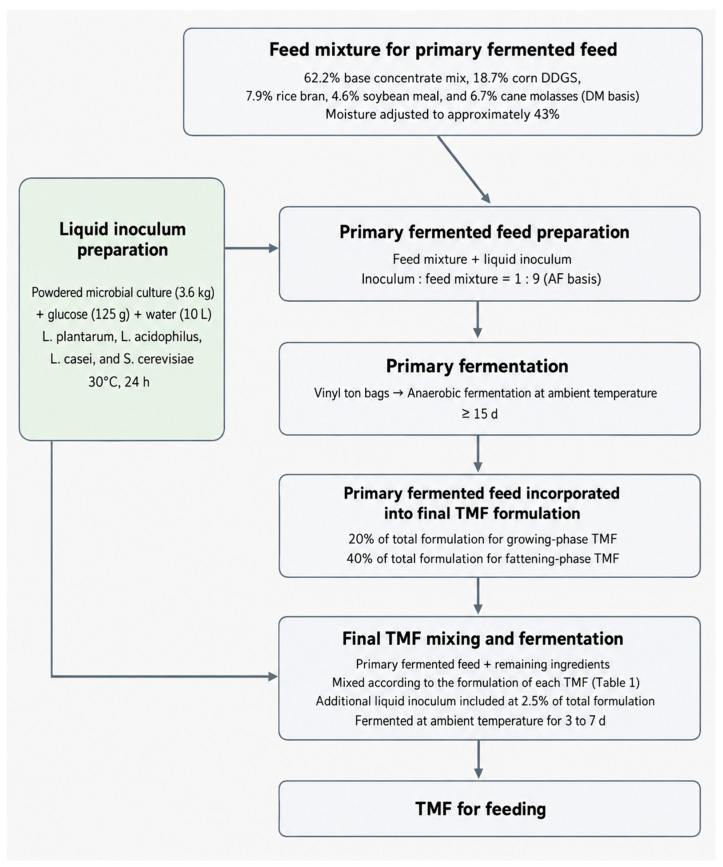
Schematic flow of total mixed fermented feed production. AF, as-fed; TMF, total mixed fermented feed.

According to the formulation of each TMF, the primary fermented feed and other feed ingredients were mixed (Table 1). The liquid inoculum was also included at 2.5% of the total formulation to ensure fermentation stability and maintain microbial activity. The total mixed feed was then fermented at ambient temperature for an additional 3 to 7 d. A schematic overview of the TMF production process is presented in Figure 1.

During the growing period (7 to 13 months of age), both groups were fed a grower TMF at 9.0–13.5 kg AF basis per day, with the feeding level gradually increased according to age. During the early fattening period (14 to 19 months of age), both the separate-feeding group and the TMF group were fed an early-fattening TMF at 14.0–15.0 kg AF/day. From the middle fattening period (20 to 25 months of age), different feeding systems were applied. The separate-feeding group received a concentrate diet for fattening cattle (9.5–10.0 kg AF/day; Table 2) and rice straw (1.74 kg AF/day) offered separately, whereas the TMF group continued to receive a middle-fattening TMF (14.5–15.0 kg AF/day). During the late fattening period (over 25 months of age to slaughter), the separate-feeding group was provided with a late-fattening concentrate (9.5 kg AF/day; Table 2) and rice straw (1.45 kg AF/day) separately, while the TMF group was fed a finisher (late-fattening) TMF at 14.0–14.5 kg AF/day, thereby receiving stage-specific TMF throughout the entire production period from growing to slaughter. The moisture and nutrient contents of all provided diets, including the TMF, were periodically analyzed. Dry matter intake was calculated by adjusting the daily feed provision amounts based on the analyzed dry matter percentages of each diet. These differences indicate that the two feeding systems have structurally distinct patterns of nutrient supply and rumen fermentation environments during the fattening period, which served as the primary basis for evaluating phenotypic differences between feeding systems. It should be noted that both groups received similar TMF-based diets during the growing and early fattening periods, and the divergence between feeding systems began from the middle fattening stage.

### 2.4. Chemical Analysis

The chemical composition of feed samples was analyzed according to the methods described by Jeon et al. [22]. The feed samples were dried at 65 °C for 72 h and ground through a cyclone mill (Foss, Hillerød, Denmark) fitted with a 1 mm screen. The nutrient composition of the feed samples was analyzed at Cumberland Valley Analytical Services Inc. (Hagerstown, MD, USA). The content of DM (#934.15), crude protein (#990.03), EE (#920.39), acid detergent fiber (#973.18), and ash (#942.05) was determined. Crude protein was calculated as 6.25 times the nitrogen content, and the total nitrogen was measured by the Dumas method using a Leco FP-528 Nitrogen Combustion Analyzer (Leco Inc., Saint Joseph, MI, USA). The acid detergent lignin (ADL) and neutral detergent fiber (aNDF; using heat-stable amylase and expressed inclusive of residual ash) contents were determined. The soluble protein, neutral detergent insoluble crude protein (NDICP) and acid detergent insoluble crude protein (ADICP) contents were also determined. The contents of ethanol soluble carbohydrate (ESC), starch, and macro- and micro-minerals were determined.

The total digestible nutrients (TDN) content was estimated according to the dairy NRC [23]. Based on TDN, metabolizable energy (ME), net energy for maintenance (NEm), and net energy for growth (NEg) were calculated using the equations described in the beef NRC [24]. Dietary carbohydrates and protein fractions were estimated according to Cornell Net Carbohydrate and Protein System [25] with the following modifications. For carbohydrate fractions, CA represents sugars and organic acids assumed to be equal to ESC, CB1 represents starch, CB2 represents soluble fiber and calculated as NFC—CA—CB1, CB3 represents available NDF and estimated by aNDF—NDICP minus 2.4 times ADL, and CC represents unavailable carbohydrate and estimated by 2.4 times ADL. For protein fractions, PA+B1 represents soluble protein and equals to SOLP, PB2 represents intermediate degradable CP and estimated by 100—NDICP—SOLP, PB3 represents slowly degradable fiber-bound CP and estimated by NDICP—ADICP, and PC represents unavailable CP and equals to ADICP. All the carbohydrate and protein fractions were expressed as g/kg of total carbohydrate or CP, respectively. Chemical compositions and calculated nutritional values of the experimental diets are presented in Table 3.

### 2.5. Statistical Analysis

Because of the large sample size and the broad temporal distribution of slaughter dates across seasons, batch effects were considered minimal and were therefore not included in the statistical model. Nevertheless, the feeding system and farm were confounded, and their effects could not be separated in this study.

Statistical analyses were performed using the PROC MIXED procedure of SAS 9.4 (SAS Institute Inc., Cary, NC, USA) to conduct a two-way analysis of variance (ANOVA). For each carcass trait (i.e., CWT, BFT, REA, and MBS), separate two-way ANOVAs were performed to test the effects of the EPD grade for the corresponding trait, feeding system, and their interaction on age at slaughter and the values of all carcass traits. Results for each factor were expressed as least squares means with their standard errors of the mean. When significant effects were detected, multiple comparisons among factors were conducted using the Tukey–Kramer post hoc test (*p* < 0.05).

To investigate how EPD grades for a given trait were associated with phenotypic variation in slaughter age and other carcass traits, regression-based analyses were conducted. Within each EPD analysis, grades A through D were treated as an ordinal variable and coded numerically according to genetic merit, with grade A = 4, grade B = 3, grade C = 2, and grade D = 1. Linear regression analyses were then performed to estimate the linear trend of each response variable across EPD grade levels. The slope obtained from each regression was subsequently normalized by dividing it by the mean value of the corresponding response variable, yielding a mean-normalized slope that allowed comparison across variables with different scales. A heatmap was constructed based on these mean-normalized slope values using R (R Foundation for Statistical Computing, Vienna, Austria) to visualize the magnitude and direction of associations among variables. To assess statistical significance, a t-test was performed for each regression coefficient under the null hypothesis that the slope was equal to zero. Corresponding *p*-values were calculated and presented alongside the heatmap to indicate the strength of evidence against the null hypothesis.

## 3. Results

### 3.1. Expected Progeny Difference Grades for Carcass Weight

Carcass weight showed a clear and generally monotonic response across EPD grades for CWT (A–D), indicating that genetic ranking for CWT was consistently reflected in field performance (Table 4; EPD grade effect, *p* < 0.001). Specifically, steers in grade A produced carcasses that were 45.2 kg heavier than those in grade D, corresponding to an increase of 9.7% relative to grade D.

The feeding system also influenced CWT (*p* < 0.001), with TMF feeding associated with a 14.0 kg (2.9%) greater CWT than the conventional separate feeding system (Table 4). However, no interaction between feeding system and EPD grade for CWT was observed (*p* > 0.05), suggesting that the effect of CWT EPD grade on CWT did not differ by feeding system.

Slaughter age differed across EPD grades for CWT (*p* < 0.001), with genetically superior (grade A) animals reaching slaughter earlier; the gap between grades A and D was 1.07 months (3.4% relative to grade D; Table 4). The effect of the feeding system on slaughter age was negligible (*p* = 0.987); however, a significant interaction between CWT EPD grade and feeding system was observed (*p* < 0.001). Under the conventional separate-feeding system, slaughter age increased from 30.29 months in grade A to 31.91 months in grade D, representing a range of 1.62 months. In contrast, under the TMF system, slaughter age ranged from 30.82 months in grade A to 31.35 months in grade D, corresponding to a difference of only 0.53 months. Overall, although slaughter age increased as CWT EPD grade decreased from A to D in both feeding systems, the magnitude of this increase was substantially smaller under the TMF system.

The other carcass traits also differed among groups having different EPD grades for CWT. Significant differences in these traits were also observed between the two feeding systems (*p* < 0.05; Table 4). In particular, BFT decreased linearly as the CWT EPD grade increased from D to A, while REA increased. This trend, however, was not observed for MBS. No significant interaction between feeding system and the CWT EPD grade was found for these traits (*p* > 0.05).

### 3.2. Expected Progeny Difference Grades for Backfat Thickness

Backfat thickness showed a clear and generally monotonic response across EPD grades for BFT (A–D), indicating that genetic ranking for BFT was consistently reflected in field performance (Table 5; EPD grade effect; *p* < 0.001). Specifically, steers in grade A had BFT that was 3.44 mm less than that of steers in grade D, corresponding to a 22.8% lower BFT. The feeding system also influenced BFT (*p* < 0.001), with TMF feeding associated with a 0.85 mm (6.5%) greater BFT than the conventional separate feeding system (Table 5). However, no interaction between feeding system and EPD grade for BFT was observed (*p* > 0.05), suggesting that the effect of BFT EPD grade on BFT did not differ by feeding system.

Slaughter age differed across BFT EPD grades (*p* = 0.023), with genetically superior (grade A) animals reaching slaughter earlier; the gap between grades A and D was 0.33 months (1.1% relative to grade D; Table 5). The main effect of feeding system on slaughter age was not significant (*p* = 0.276), and no significant interaction between BFT EPD grade and feeding system was detected (*p* > 0.05), indicating that the association between BFT genetic merit and slaughter age was largely consistent across feeding systems.

The other carcass traits also differed among groups having different EPD grades for BFT. Significant differences in CWT and REA were also observed between the two feeding systems (*p* < 0.001), whereas the feeding system effect on MBS was not significant (*p* = 0.061; Table 5). In particular, CWT and ribeye area decreased as the BFT EPD grade declined from A to D. This trend, however, was less consistent for MBS. No significant interaction between feeding system and the BFT EPD grade was found for these traits (*p* > 0.05).

### 3.3. Expected Progeny Difference Grades for Ribeye Area

Ribeye area showed a clear and generally monotonic response across EPD grades for REA (A–D), indicating that genetic ranking for REA was consistently reflected in field performance (Table 6; EPD grade effect; *p* < 0.001). Specifically, steers in grade A had an REA that was 10.77 cm^2^ (10.9%) greater than that of steers in grade D. The feeding system also influenced REA (*p* < 0.001), with TMF feeding associated with a 2.42 cm^2^ (2.4%) greater REA than the conventional separate feeding system (Table 6). However, no interaction between feeding system and EPD grade for REA was observed (*p* > 0.05), suggesting that the effect of REA EPD grade on REA did not differ by feeding system.

Slaughter age differed across REA EPD grades (*p* < 0.001), with genetically superior (grade A) animals reaching slaughter earlier; the gap between grades A and D was 0.82 months (2.6% relative to grade D; Table 6). The main effect of feeding system on slaughter age was not significant (*p* = 0.357), and no significant interaction between REA EPD grade and feeding system was detected (*p* > 0.05), indicating that the association between REA genetic merit and slaughter age was consistent across feeding systems.

The other carcass traits also differed among groups having different EPD grades for REA. Significant differences in these traits were also observed between the two feeding systems (*p* < 0.05; Table 6). In particular, CWT increased, whereas BFT decreased, as the REA EPD grade increased from D to A. MBS also tended to be greater in genetically superior groups, although this trend was less distinct than that observed for REA itself. No significant interaction between feeding system and the REA EPD grade was found for these traits, although the interaction for BFT approached significance (*p* = 0.064).

### 3.4. Expected Progeny Difference Grades for Marbling Score

Marbling score showed a clear and generally monotonic response across EPD grades for MBS (A–D), indicating that genetic ranking for MBS was consistently reflected in field performance (Table 7; EPD grade effect; *p* < 0.001). Specifically, steers in grade A had marbling scores that were 1.57 units (26.8%) greater than those of steers in grade D. The feeding system also influenced MBS (*p* = 0.018), with TMF feeding associated with a 0.14-unit (2.1%) greater MBS than the conventional separate feeding system (Table 7). However, no interaction between feeding system and EPD grade for MBS was observed (*p* > 0.05), suggesting that the effect of MBS EPD grade on MBS did not differ by feeding system.

Slaughter age differed across MBS EPD grades (*p* = 0.001), with genetically superior (grade A) animals reaching slaughter earlier; the gap between grades A and D was 0.37 months (1.2% relative to grade D; Table 7). The main effect of feeding system on slaughter age was not significant (*p* = 0.323), and no significant interaction between MBS EPD grade and feeding system was detected (*p* > 0.05), indicating that the association between MBS genetic merit and slaughter age was consistent across feeding systems.

The other carcass traits also differed among groups having different EPD grades for MBS. Significant differences in these traits were also observed between the two feeding systems (*p* < 0.05; Table 7). In particular, CWT and REA decreased as the MBS EPD grade declined from A to D. This trend, however, was not consistently observed for BFT, which showed a non-monotonic pattern across grades. No significant interaction between feeding system and the MBS EPD grade was found for these traits (*p* > 0.05).

### 3.5. Integrated Interpretation Across Slaughter Age and Carcass Traits

**Figure 2:**
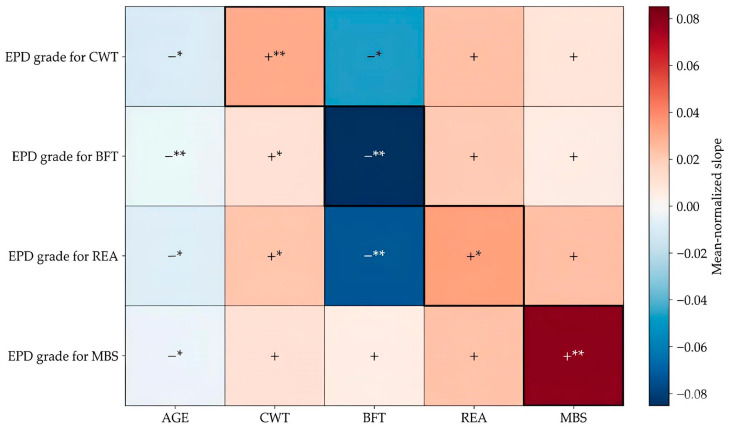
Heatmap summarizing mean-normalized linear slopes of slaughter age and carcass traits across expected progeny difference (EPD) grades in Hanwoo steers. For each EPD analysis, grades were coded according to genetic merit, with grade A = 4, grade B = 3, grade C = 2, and grade D = 1. Linear regression was performed to estimate the slope of each response variable across grade levels, and each slope was normalized by dividing it by the mean value of the corresponding response variable to allow comparison across variables with different scales. Red indicates a positive mean-normalized slope, whereas blue indicates a negative mean-normalized slope. Positive slopes indicate that the response variable increased with greater EPD genetic merit, whereas negative slopes indicate that the response variable decreased with greater EPD genetic merit. The symbols + and − denote the direction of the slope. Asterisks indicate the significance of the regression slope (* *p* < 0.05, ** *p* < 0.01), whereas cells without asterisks indicate non-significant slopes (*p* ≥ 0.05). Thick borders indicate the target carcass trait corresponding to each EPD analysis.

To facilitate an integrative comparison of multi-trait responses, Figure 2 summarizes the mean-normalized linear trends of slaughter age and carcass traits across EPD grades. Positive values indicate that the corresponding response variable tended to increase as EPD grade increased from D to A, whereas negative values indicate the opposite pattern. The heatmap showed that slaughter age generally decreased as genetic merit increased across all four EPD analyses, with significant negative slopes observed in each analysis. CWT generally increased with improving genetic merit across EPD analyses, although this trend was not significant in the MBS EPD analysis. BFT decreased with improving genetic merit in the CWT, BFT, and REA EPD analyses, whereas no significant negative trend was observed in the MBS EPD analysis. No significant linear trend was observed for REA or MBS in relation to EPD grade, except within their respective trait-specific EPD analyses. Overall, this visualization indicates that the direction and relative magnitude of grade-associated changes differed among response variables and EPD analyses, while also highlighting partial overlap among slaughter age and carcass traits.

## 4. Discussion

In the Hanwoo industry, EPD is widely used as a key indicator for predicting genetic potential for major carcass traits; however, the actual expression of this genetic potential may be modulated by environment, particularly the feeding system during the fattening period. Although previous studies have reported the effects of genetic merit or feeding system individually, few have simultaneously evaluated the relationship between these two factors using large-scale field data. In this context, the present study was conducted using a large field dataset (5037 steers) to quantitatively examine the relationships between Hanwoo EPDs and major carcass traits and to evaluate how the fattening feeding system (separate feeding vs. TMF) influences the phenotypic expression of genetic potential.

In the present study, CWT, REA, BFT, and MBS all differed significantly according to the EPD grades for their respective traits (*p* < 0.001). Although most traits exhibited consistent and monotonic trends across EPD grades, a quadratic pattern was observed for MBS across CWT EPD grades (Table 4). Relatively higher MBSs were observed in the lowest CWT EPD group (grade D) compared with grades B and C. Although the underlying mechanism of this phenomenon cannot be fully elucidated based on the present data, one possible explanation is related to differences in genetically determined mature body weight. Specifically, animals with smaller body size tend to reach mature body weight earlier. Consequently, their metabolic processes may shift more favorably toward fat deposition during the latter fattening phase, which may ultimately promote marbling development [26]. However, this explanation does not fully account for the non-monotonic ranking pattern observed among grades, and therefore the result should be interpreted cautiously.

Nevertheless, the overall statistically significant differences in carcass traits among EPD grades indicate that EPD can be used as an indicator of carcass traits at slaughter in Hanwoo cattle, which is generally consistent with findings from previous studies conducted in this breed [27,28]. The particularly clear separation among EPD grades for highly heritable traits such as CWT and MBS further supports the robustness of EPD-based selection in reflecting actual field performance [3].

Figure 2 serves as a complementary visualization of the direction and relative magnitude of trait-wise linear trends across EPD grades. Unlike the table-based comparisons, which focused on differences among discrete grade groups, the heatmap summarized whether slaughter age and carcass traits tended to decrease or increase as EPD genetic merit increased from grade D to grade A. This visualization was intended to complement, rather than replace, the formal mixed-model comparisons presented in Table 4, Table 5, Table 6 and Table 7.

The heatmap showed that slaughter age generally decreased as EPD genetic merit increased across analyses, whereas CWT tended to increase. BFT displayed negative trends under the CWT, BFT, and REA EPD analyses, while MBS showed the clearest positive trend under the MBS EPD analysis. Several non-target variables exhibited weaker or non-significant trends, indicating that the phenotypic expression associated with EPD grade varied in both strength and direction depending on the response variable considered. These findings suggest that EPD-related responses in Hanwoo steers are partly trait-specific but also reflect partial overlap among slaughter age and carcass characteristics.

The feeding system exhibited a significant main effect on most major carcass traits, with the TMF-fed group generally showing slightly superior carcass performance compared with the separately fed group. These results may be associated with the ability of TMF to reduce selective feeding through improved physical uniformity of the diet and to maintain a more stable rumen fermentation environment, thereby enhancing nutrient utilization efficiency [9,29,30].

In addition, in the case of TMF, the progressive improvement in digestive and metabolic efficiency during the long fattening period, driven by organic acid production and stabilization of microbial communities, may also have contributed to the observed advantages [13,31].

Because actual feed intake was not measured in the present field study, nutrient intake could not be directly quantified. Therefore, the following interpretation is based on the programmed feed allowance and analyzed diet composition rather than on observed intake. During the middle fattening period, the TMF group was estimated to receive 9.45–9.78 kg DM/day, 1.70–1.76 kg CP/day, 3.81–3.94 kg aNDF/day, 2.76–2.86 kg NFC/day, 1.41–1.46 kg starch/day, 0.51–0.53 kg EE/day, 6.43–6.65 kg TDN/day, 98.3–101.7 MJ ME/day, and 40.7–42.1 MJ NEg/day, whereas the separate-feeding group was estimated to receive 9.78–10.22 kg DM/day, 1.55–1.63 kg CP/day, 3.04–3.15 kg aNDF/day, 4.15–4.36 kg NFC/day, 3.27–3.44 kg starch/day, 0.37–0.39 kg EE/day, 6.87–7.20 kg TDN/day, 102.0–106.9 MJ ME/day, and 43.6–45.8 MJ NEg/day. During the late fattening period, the corresponding values were 8.82–9.14 vs. 9.48 kg DM/day, 1.51–1.56 vs. 1.48 kg CP/day, 3.38–3.50 vs. 2.94 kg aNDF/day, 2.87–2.97 vs. 3.93 kg NFC/day, 1.64–1.70 vs. 2.88 kg starch/day, 0.56–0.58 vs. 0.47 kg EE/day, 6.17–6.39 vs. 6.73 kg TDN/day, 93.5–96.8 vs. 100.0 MJ ME/day, and 39.7–41.1 vs. 43.1 MJ NEg/day for TMF and separate feeding, respectively. Thus, under the programmed feeding scheme, TMF supplied relatively more CP, aNDF, and EE, whereas separate feeding supplied more NFC, starch, and energy during the fattening period. These results suggest that differences in carcass performance between feeding systems may have been associated more with differences in the composition and form of nutrient supply than with energy density alone.

Notably, the programmed nutrient supply did not indicate a clear energy advantage of TMF over separate feeding during the fattening period. In fact, calculated TDN, ME, and NEg supply tended to be comparable or slightly greater under the separate-feeding system. Therefore, the greater carcass weight observed in the TMF-fed cattle is unlikely to be explained simply by a higher planned energy supply. Rather, the advantage of TMF may be related to differences in the actual utilization of nutrients after feed delivery. Because TMF provides forage and concentrate in a physically uniform and fermented form, it may reduce selective feeding, stabilize ruminal fermentation, and improve the digestibility and effective availability of nutrients to the animal. Previous studies have suggested that mixed and fermented feeding systems can enhance intake uniformity, rumen stability, and the digestibility of starch and fiber, thereby improving the efficiency with which nutrients are converted into growth and carcass deposition [9,13,14]. In this context, the superior carcass weight of the TMF-fed group may reflect not only the nutrient composition of the diet itself but also a greater amount of nutrients actually digested and utilized by the animal. This interpretation should be made cautiously, however, because actual intake and digestibility were not measured in the present study.

For most carcass traits, the interaction between EPD grade and feeding system was not significant, indicating that the relative ranking among EPD grades was generally maintained regardless of differences in feeding system. This finding suggests that the feeding system primarily influenced the absolute level of carcass performance within a given genetic potential rather than altering the genetic ranking of animals. This finding is broadly consistent with the concept that favorable management conditions may influence the level of phenotypic expression without necessarily causing reranking of genetic merit [2].

Slaughter age was consistently shorter for grade A and longer for grade D across all EPD categories, indicating that genetically superior animals tended to reach target carcass performance more rapidly. This pattern implies that differences in growth efficiency and energy utilization are reflected in slaughter age, suggesting that EPDs may serve not only as indicators of carcass merit but also as useful references for production efficiency.

Meanwhile, a significant interaction between CWT EPD grade and feeding system was observed for slaughter age (*p* < 0.001), even though the overall main effect of feeding system was not significant. Specifically, the difference in slaughter age between grades A and D was markedly smaller under TMF than under separate feeding. This result suggests that TMF may have reduced the delay in reaching the practical slaughter endpoint among steers with lower CWT genetic merit. In commercial farm conditions, slaughter timing is determined not by carcass weight alone but by an overall management decision reflecting age, finishing status, and farm-specific marketing strategy. Therefore, the presence of an interaction for slaughter age, despite the absence of a corresponding interaction for carcass weight, is not necessarily contradictory. One possible explanation is that TMF, by providing a more uniform mixture of forage and concentrate, may reduce variation in nutrient intake and stabilize the feeding environment, thereby lessening the extent to which lower-growth animals are delayed in reaching a commercially acceptable finishing stage [9,10]. Under separate feeding, by contrast, variation in nutrient intake and feeding behavior may be more strongly expressed, which could amplify differences in slaughter timing among genetic groups [32,33]. This interpretation should be made cautiously, however, because the present dataset did not include direct measurements of individual intake, feeding behavior, or the specific criteria used to determine slaughter timing.

It should be noted that this study was based on observational field data collected from commercial farms, and animals were not randomly assigned to feeding systems. Therefore, potential confounding factors associated with farm-level management, environmental variation, or unmeasured nutritional differences cannot be completely excluded. In addition, because the nutrient composition of the diets differed between feeding systems, the effects of feeding system per se could not be completely separated from those of diet composition. Accordingly, the present results should be interpreted as reflecting the combined influence of feeding system and associated nutritional characteristics under practical farm conditions. Nevertheless, the large sample size and the consistency of the observed trends across multiple traits support the robustness of the overall conclusions.

## 5. Conclusions

Based on a large dataset collected under practical production conditions, carcass trait performance was significantly associated with EPD grade in Hanwoo steers. Furthermore, TMF improved carcass traits compared with separate feeding, although it did not override genetic potential. Although a substantial proportion of variation in carcass trait expression cannot be explained by these two factors alone, incorporating Hanwoo EPD grades together with adopting a TMF feeding system can be a beneficial strategy for producing high-quality carcasses in Hanwoo production.

## Figures and Tables

**Table 1:** Diet formulation of the total mixed fermented feed (% dry matter).

Ingredients	Diets ^1^			
	Growing	Early Fattening	Middle Fattening	Late Fattening
Italian rye grass silage	13.03			
Alfalfa hay	10.44			
Timothy hay	4.53			
Barley straw	6.10	13.42	12.30	9.78
Oat hay	4.43	4.46	2.97	2.97
Fermented feed ^2^	18.29	36.76	36.75	36.74
Base concentrate mix ^3^	16.94	17.03	14.19	14.18
Steam flaked Corn		4.72	9.33	14.01
Soybean meal	2.10	1.41	1.41	
Corn DDGS ^4^	8.28	1.97	1.60	
Cottonseed whole		4.46	5.94	7.43
Spent mushroom substrate	9.23	6.63	6.62	6.62
Wet brewers grains	2.32	1.94	1.94	1.94
Soybean curd residue		1.96	1.96	1.96
Cane molasses	2.42	2.43	2.43	2.43
Fermentation starters ^5^	0.43	0.43	0.43	0.43
Vitamin–mineral premix ^6^	0.30	0.15	0.15	0.22
Others	1.16	2.23	1.98	1.29

^1^ Growing, 7~13 months of age; early fattening, 14~19 months of age; middle fattening, 20~25 months of age; late fattening, >25 months of age. ^2^ Base concentrate mix, 62.2; soybean meal, 4.6; corn DDGS, 18.7; rice bran, 7.9; cane molasses, 6.7. Values are expressed on a dry matter basis. ^3^ Corn grain (fine), 28.9; soybean meal, 6.9; corn DDGS, 15.0; palm kernel meal, 10.1; corn gluten feed, 20.8; wheat bran, 9.8; rice bran, 5.0; cane molasses, 1.8; and others, 1.7. Values are expressed on a dry matter basis. ^4^ Dried distillers grains with solubles. ^5^ Fermentation starters: Lactobacillus plantarum (9.0 × 10^11^ CFU/g), Lactobacillus acidophilus (9.8 × 10^10^ CFU/g), Lactobacillus casei (6.0 × 10^11^ CFU/g), Saccharomyces cerevisiae (3.0 × 10^10^ CFU/g). ^6^ Vitamin–mineral premix: 10,000 IU/g vitamin A, 1150 IU/g vitamin D, 20,000 IU/kg vitamin E, 48,000 mg/kg Mn, 70,000 mg/kg Zn, 26,000 mg/kg Fe, 10,000 mg/kg Cu, 1000 mg/kg I, 530 mg/kg Co.

**Table 2:** Diet formulation of the experimental concentrate mixes (% dry matter).

Ingredients	Concentrate Mix ^1^	
	Middle Fattening	Late Fattening
Steam flaked Corn	26.20	25.90
Wheat fine	11.23	11.22
Corn fine	-	1.27
Wheat flour	17.77	17.75
Soybean meal	0.99	0.99
Corn DDGS ^2^	11.76	6.35
Palm kernel meal	7.00	0.89
Copra meal	2.03	2.03
Corn gluten feed	15.62	15.60
Cottonseed hulls	1.04	1.03
Soy hulls	0.56	7.76
Rice bran	-	3.02
Cane molasses	1.45	1.45
Sodium bicarbonate	1.10	1.00
Limestone fine	0.93	0.83
Salt	0.23	0.23
Vitamin–mineral premix ^3^	0.28	0.65
Others	1.81	2.03

^1^ Middle fattening, 20~25 months of age; late fattening, >25 months of age. ^2^ Dried distillers grains with solubles. ^3^ Vitamin–mineral premix: 10,000 IU/g vitamin A, 1150 IU/g vitamin D, 20,000 IU/kg vitamin E, 48,000 mg/kg Mn, 70,000 mg/kg Zn, 26,000 mg/kg Fe, 10,000 mg/kg Cu, 1000 mg/kg I, 530 mg/kg Co.

**Table 3:** Analyzed chemical composition (g/kg DM or as stated) of the experimental diets.

Items ^1^	TMF ^2^				Concentrate Mix		Rice Straw
	Growing	Early Fattening	Middle Fattening	Late Fattening	Middle Fattening	Late Fattening	
DM, g/kg as fed	652	625	652	630	872	867	859
OM	895	896	907	921	920	918	855
CP	175	178	180	171	175	169	68
SOLP	64	66	61	63	51	55	28
NDICP	23	21	22	21	21	20	18
ADICP	16	15	16	19	13	15	14
CF	147	157	157	142	53	59	318
aNDF	466	403	403	383	243	253	688
ADF	278	217	214	220	98	117	482
Lignin	53	39	41	49	26	33	76
Starch	55	132	149	186	392	348	14
EE	50	66	54	63	41	54	20
Ash	106	104	93	79	80	82	145
Ca	14	16	15	12	11	10	2
P	6	7	7	7	6	6	1
Mg	4	5	5	4	5	6	2
K	15	13	13	12	10	10	11
S	3	4	3	3	3	3	2
Na	2	2	1	1	4	4	2
Cl	5	5	4	4	7	6	7
TDN, % DM	62.5	68.8	68.1	69.9	75.4	75.5	44.2
ME, MJ/kg	9.8	10.5	10.4	10.6	11.2	11.2	6.2
NEm, MJ/kg	6.1	6.9	6.8	7.0	7.7	7.7	3.1
NEg, MJ/kg	3.6	4.4	4.3	4.5	5.1	5.1	0.9
Carbohydrates	670	652	673	687	705	695	768
NFC	227	270	292	325	483	462	97
Carbohydrate fractions, g/kg carbohydrate							
CA	65	83	74	43	116	118	30
CB1	83	202	221	271	556	501	18
CB2	190	129	138	159	13	46	78
CB3	471	443	422	358	227	222	636
CC	190	143	144	169	88	113	237
Protein fractions, g/kg CP							
PA+PB1	364	371	339	368	291	325	412
PB2	501	513	541	510	588	555	331
PB3	43	30	32	11	45	31	53
PC	92	86	88	112	76	88	204

^1^ DM: dry matter, OM: organic matter, CP: crude protein, SOLP: soluble CP, NDICP: neutral detergent insoluble CP, ADICP: acid detergent insoluble CP, aNDF: neutral detergent fiber analyzed using a heat-stable amylase and expressed inclusive of residual ash, ADF: acid detergent fiber, TDN: total digestible nutrients, ME: metabolizable energy, NEm: net energy for maintenance, NEg: net energy for growth, NFC: non-fiber carbohydrate, CA: carbohydrate A fraction; ethanol soluble carbohydrates, CB1: carbohydrate B1 fraction; starch, CB2: carbohydrate B2 fraction; soluble fiber, CB3: carbohydrate B3 fraction; available insoluble fiber, CC: carbohydrate C fraction; unavailable carbohydrate, PA+PB1: protein A and B1 fractions; soluble CP, PB2: protein B2 fraction; intermediate degradable CP, PB3: protein B3 fraction; slowly degradable fiber-bound CP, PC: protein C fraction; unavailable CP. ^2^ TMF, Total mixed fermented feed.

**Table 4:** Effects of expected progeny difference grade for carcass weight and feeding system on age at slaughter and carcass traits in Hanwoo steers.

Items ^1^	EPD Grade for CWT ^2^				SEM	Feeding System ^3^		SEM	*p*-Value ^4^		
	A	B	C	D		TMF	CON		EPD	Diet	Interaction
AGE, months	30.56 ^c^	30.86 ^b^	31.02 ^b^	31.63 ^a^	0.102	31.02	31.02	0.066	<0.001	0.987	<0.001
CWT, kg	509.76 ^a^	490.68 ^b^	478.58 ^c^	464.59 ^d^	2.310	492.89	478.91	1.498	<0.001	<0.001	0.269
BFT, mm	12.23 ^c^	13.32 ^b^	13.80 ^ab^	14.17 ^a^	0.209	13.83	12.93	0.135	<0.001	<0.001	0.544
REA, cm^2^	108.09 ^a^	102.95 ^b^	101.41 ^bc^	100.26 ^c^	0.594	104.24	102.11	0.385	<0.001	<0.001	0.607
MBS, unitless	6.95 ^a^	6.49 ^b^	6.42 ^b^	6.76 ^a^	0.079	6.73	6.58	0.051	<0.001	0.018	0.410

^1^ AGE, age at slaughter; CWT, carcass weight; BFT, backfat thickness; REA, ribeye area; MBS, marbling score. ^2^ EPD, expected progeny difference; CWT, carcass weight. ^3^ TMF, total mixed fermented feed; CON, conventional system in which forage and concentrate were fed separately. ^4^ EPD, expected progeny difference for carcass weight. ^a–d^ Means that do not have common superscripts differ significantly within the treatments (*p* < 0.05).

**Table 5:** Effects of expected progeny difference grade for backfat thickness and feeding system on age at slaughter and carcass traits in Hanwoo steers.

Items ^1^	EPD Grade for BFT ^2^				SEM	Feeding System ^3^		SEM	*p*-Value ^4^		
	A	B	C	D		TMF	CON		EPD	Diet	Interaction
AGE, months	30.76 ^b^	30.89 ^ab^	30.96 ^ab^	31.09 ^a^	0.086	30.97	30.88	0.064	0.023	0.276	0.515
CWT, kg	498.42 ^a^	490.26 ^b^	487.52 ^bc^	481.75 ^c^	1.988	496.66	482.31	1.484	<0.001	<0.001	0.699
BFT, mm	11.63 ^d^	12.95 ^c^	14.05 ^b^	15.07 ^a^	0.170	13.85	13.00	0.127	<0.001	<0.001	0.308
REA, cm^2^	107.56 ^a^	103.16 ^b^	101.99 ^bc^	101.06 ^c^	0.497	104.53	102.36	0.371	<0.001	<0.001	0.740
MBS, unitless	6.80 ^a^	6.58 ^b^	6.55 ^b^	6.69 ^ab^	0.066	6.71	6.60	0.049	0.006	0.061	0.338

^1^ AGE, age at slaughter; CWT, carcass weight; BFT, backfat thickness; REA, ribeye area; MBS, marbling score. ^2^ EPD, expected progeny difference; BFT, backfat thickness. ^3^ TMF, total mixed fermented feed; CON, conventional system in which forage and concentrate were fed separately. ^4^ EPD, expected progeny difference for backfat thickness. ^a–d^ Means that do not have common superscripts differ significantly within the treatments (*p* < 0.05).

**Table 6:** Effects of expected progeny difference grade for ribeye area and feeding system on age at slaughter and carcass traits in Hanwoo steers.

Items ^1^	EPD Grade for REA ^2^				SEM	Feeding System ^3^		SEM	*p*-Value ^4^		
	A	B	C	D		TMF	CON		EPD	Diet	Interaction
AGE, months	30.61 ^c^	30.85 ^bc^	30.96 ^b^	31.43 ^a^	0.090	31.00	30.93	0.064	<0.001	0.357	0.118
CWT, kg	507.45 ^a^	490.53 ^b^	481.21 ^c^	473.97 ^d^	2.067	495.81	480.76	1.460	<0.001	<0.001	0.563
BFT, mm	12.00 ^d^	12.90 ^c^	13.77 ^b^	14.92 ^a^	0.182	13.85	12.95	0.129	<0.001	<0.001	0.064
REA, cm^2^	109.75 ^a^	103.19 ^b^	100.88 ^c^	98.98 ^d^	0.513	104.41	101.99	0.362	<0.001	<0.001	0.233
MBS, unitless	7.09 ^a^	6.45 ^b^	6.44 ^b^	6.56 ^b^	0.069	6.71	6.56	0.049	<0.001	0.011	0.486

^1^ AGE, age at slaughter; CWT, carcass weight; BFT, backfat thickness; REA, ribeye area; MBS, marbling score. ^2^ EPD, expected progeny difference; REA, ribeye area. ^3^ TMF, total mixed fermented feed; CON, conventional system in which forage and concentrate were fed separately. ^4^ EPD, expected progeny difference for ribeye area. ^a–d^ Means that do not have common superscripts differ significantly within the treatments (*p* < 0.05).

**Table 7:** Effects of expected progeny difference grade for marbling score and feeding system on age at slaughter and carcass traits in Hanwoo steers.

Items ^1^	EPD Grade for MBS ^2^				SEM	Feeding System ^3^		SEM	*p*-Value ^4^		
	A	B	C	D		TMF	CON		EPD	Diet	Interaction
AGE, months	30.74 ^c^	30.80 ^bc^	31.05 ^ab^	31.11 ^a^	0.084	30.96	30.89	0.063	0.001	0.323	0.284
CWT, kg	502.43 ^a^	486.60 ^b^	483.44 ^b^	485.61 ^b^	1.939	496.86	482.18	1.466	<0.001	<0.001	0.851
BFT, mm	13.03 ^b^	13.58 ^a^	13.26 ^ab^	12.89 ^b^	0.172	13.62	12.76	0.130	0.011	<0.001	0.721
REA, cm^2^	108.20 ^a^	103.46 ^b^	101.57 ^c^	100.86 ^c^	0.485	104.63	102.42	0.367	<0.001	<0.001	0.401
MBS, unitless	7.43 ^a^	6.75 ^b^	6.30 ^c^	5.86 ^d^	0.062	6.66	6.52	0.047	<0.001	0.018	0.199

^1^ AGE, age at slaughter; CWT, carcass weight; BFT, backfat thickness; REA, ribeye area; MBS, marbling score. ^2^ EPD, expected progeny difference; MBS, marbling score. ^3^ TMF, total mixed fermented feed; CON, conventional system in which forage and concentrate were fed separately. ^4^ EPD, expected progeny difference for marbling score. ^a–d^ Means that do not have common superscripts differ significantly within the treatments (*p* < 0.05).

## Data Availability

Data can be made available from the authors upon request.

## Abbreviations

The following abbreviations are used in this manuscript:

ADG	Average daily gain
ADICP	Acid detergent insoluble crude protein
ADL	Acid detergent lignin
AF	As-fed
aNDF	Neutral detergent fiber
ANOVA	Analysis of variance
BFT	Backfat thickness
CP	Crude protein
CWT	Carcass weight
DM	Dry matter
EBV	Estimated breeding value
EE	Ether extract
EPD	Expected progeny difference
ESC	Ethanol soluble carbohydrate
gEBV	Genomic estimated breeding value
G×E	Genotype × environment
LAB	Lactic acid-producing bacteria
MBS	Marbling score
ME	Metabolizable energy
NDICP	Neutral detergent insoluble crude protein
NEg	Net energy for growth
NEm	Net energy for maintenance
REA	Ribeye area
TDN	Total digestible nutrients
TMF	Total mixed fermented feed
TMR	Total mixed ration
